# Long-Term Usage of Oral Glucocorticoids Leading to Adrenal Insufficiency: A Comprehensive Review of the Literature

**DOI:** 10.7759/cureus.38948

**Published:** 2023-05-12

**Authors:** Ana G Monge Chacón, Chen Wang, Danish Waqar, Saba Amreen Syeda, Rohan Kumar, D Ragasri Meghana

**Affiliations:** 1 Medicine and Surgery, Universidad Hispanoamericana, Escazu, CRI; 2 Medicine, China Medical University, Taichung, TWN; 3 Internal Medicine, Nephrology, Loyola University Medical Center, Chicago, USA; 4 Medicine, Deccan College of Medical Sciences, Plano, USA; 5 Medicine, Pandit Bhagwat Dayal Sharma Post Graduate Institute of Medical Sciences, Rohtak, IND; 6 Medicine, Kakatiya Medical College, Warangal, IND

**Keywords:** adrenal insufficiency, corticosteroids, oral glucocorticoids, adrenal suppression, primary adrenal insufficiency

## Abstract

Systemic glucocorticoid therapy is used worldwide by one to three percent of the general population and 0.5-1.8% on long-term oral glucocorticoid use. It is widely used in conditions such as inflammation, autoimmune diseases, and cancer to inhibit inflammatory responses. One of the possible undesirable side effects of exogenous corticosteroid treatment is adrenal suppression upon discontinuation of the medication and adrenal insufficiency after utilizing the supraphysiologic doses for more than one month. To prevent patients from the unwanted signs and symptoms of adrenal insufficiency, including fatigue, gastrointestinal upset, anorexia/weight loss, etc., better management of the quantity and frequency of exogenous corticosteroid use, as well as better education before starting its use, is needed. For patients actively on exogenous corticosteroids, a close follow-up must be in place to avoid adrenal suppression after the eventual discontinuation of their use. This review article summarizes the important studies to date on this subject, especially oral glucocorticoid use, and analyzes risks such as dose, duration of exposure, and comorbidities of adrenal insufficiency associated with oral glucocorticoid use. We comprehensively include information on those with primary adrenal insufficiency and pediatric patients, hoping to provide better insight and clinical reference.

## Introduction and background

Adrenal insufficiency (AI) is a life-threatening disorder requiring prompt diagnosis and management. Patients with adrenal insufficiency often present with fatigue, anorexia, nausea or vomiting, unintentional weight loss, postural hypotension, and recurrent muscle and abdominal pain [1-3]. There are several causes of adrenal insufficiency, including different pathologies of the adrenal gland (primary adrenal insufficiency, PAI), hypothalamic or pituitary pathology (secondary adrenal insufficiency, SAI), or suppression of the hypothalamic-pituitary-adrenal (HPA) axis by exogenous glucocorticoids (GCs) [2,4,5]. 

In the normal hypothalamic-pituitary-adrenal (HPA) axis, the anterior pituitary releases adrenocorticotropic hormone (ACTH) in response to hypothalamic corticotropin‐releasing hormone (CRH) stimulation. ACTH is produced by the cleavage of the precursor polypeptide proopiomelanocortin (POMC) by proprotein convertase-1 (PC1). ACTH stimulates the adrenal glands zona fasciculata and zona reticularis to produce glucocorticoids and androgens, respectively [6]. Primary adrenal insufficiency is characterized by insufficient production of aldosterone and hydrocortisone from the adrenal glands. Addison's disease is an acquired primary adrenal insufficiency. It is most commonly due to autoimmune destruction of the adrenal cortex by autoantibodies against the steroid 21-hydroxylase enzyme [7]. Other causes of primary adrenal insufficiency include congenital adrenal hyperplasia (CAH), adrenal hemorrhage, infections, cancer, and certain drugs [8]. Secondary or central adrenal insufficiency (CAI) is defined as the impairment of pituitary hormone synthesis and release of ACTH, as well as an impaired release or action of CRH from the hypothalamus when caused by disease or injury to the hypothalamic-pituitary area, or when prolonged exogenous glucocorticoid administration exceeding physiological replacement doses. Cortisol production and release are therefore reduced in the absence of ACTH or CRH in secondary adrenal insufficiency. As mineralocorticoids are primarily regulated by the renin-angiotensin system and only partially by ACTH, they are generally spared in CAI, whereas they are impaired in primary adrenal insufficiency [9]. 

Glucocorticoid treatment has been extensively used in clinical practice, such as treating autoimmune diseases, inflammation, and cancer to inhibit inflammatory responses [10-12]. It may cause adrenal insufficiency via feedback suppression of corticotropin-releasing hormone (CRH) and adrenocorticotropic hormone (ACTH), eventually inducing adrenocortical hypoplasia and atrophy and rendering the HPA axis unable to produce an adequate cortisol response to stress [13-15]. Administration routes of glucocorticoids include intravenous, oral, and local, such as inhaled, topical, and intra-articular joint injections. Multiple administration modes are often used [16]. The prevalence of oral glucocorticoids ranges from 0.5 to 1.8% in the general population, more commonly used by women and the elderly, and long-term use (more than three months) is 1% [17]. Studies have shown that around half of the patients develop signs and symptoms of adrenal insufficiency during oral glucocorticoid treatments or shortly after withdrawal [18]. However, the detection of glucocorticoid-induced adrenal insufficiency is often based on clinical suspicion rather than routine testing. Many cases were under-recognized and were waited until developing as acute crises at emergent admission [19]. Therefore, the importance of identifying patients on long-term oral glucocorticoids at risk of adrenal insufficiency cannot be over-emphasized. We see the need for a comprehensive review to provide references and raise awareness of this issue.

We have reviewed published research articles in peer-reviewed journals from 1998 to 2023 on Pubmed, Google Scholar, and Medline. Through our review, we aim to gain a deeper insight into various epidemiological and prognostic factors on the use of long-term oral glucocorticoids and, consequently, the development of adrenal insufficiency. This review article provides an approach to identifying risk factors such as doses, duration of glucocorticoid use, certain medications, genetic variations in patients, and comorbidities that can help risk stratification of patients. Identifying patients at high risk for developing adrenal insufficiency is crucial to avoid unnecessary prolonged glucocorticoid treatments [20]. The population of our review includes those with primary adrenal insufficiency, glucocorticoid-induced adrenal insufficiency, and pediatric patients. 

## Review

Methods and results 

A total of 95 articles were read, analyzed, and found to be directly associated with the subject being reviewed. Long-term use of glucocorticoids can result in adrenal insufficiency, especially when abruptly discontinuing the medication; nevertheless, we also see many poor outcomes for patients who have primary adrenal insufficiency and are treated with long-term glucocorticoids. Therefore, we will be looking at these results in both instances. 

Patients with adrenal insufficiency being treated with oral glucocorticoids

As illustrated in Table 1, a group of Italian researchers studied the outcomes of two groups of patients with adrenal insufficiency [21]. Group A on conventional glucocorticoids like cortisone acetate or hydrocortisone, and group B on dual-release hydrocortisone (DR-HC). Both groups were studied at varying doses. They analyzed data from 100 patients with different types of adrenal insufficiency, all seen at the University of Palermo in their endocrinology division. During the study, it was found that group B (on DR-HC) had better general outcomes regarding adrenal insufficiency crises, as this group had no patients present any instances of adrenal suppression. They also found that group B had a higher prevalence of osteoporosis and/or osteopenia, diabetes, lower sodium, and higher levels of HbA1c. These two groups were studied for 48 months to see the varying details of their comorbidities. The results showed that patients on long-term DR-HC had a decrease in metabolic parameters like BMI, waist circumference (WC), diastolic blood pressure (DBP), total cholesterol (TC), LDL-C, and HbA1C and improvement in cardiovascular health as opposed to conventional glucocorticoids that cause worsening of such parameters, irrespective of the dosage used.

**Table 1:** Anthropometric and metabolic parameters in groups A and B at baseline and after 48 months of treatment Pa is a comparison between baseline and after 48 months in group A; Pb is a comparison between baseline and after 48 months in group B. WC - waist circumference, HDL - high-density lipoprotein, LDL - low-density lipoprotein Reproduced under the terms of the Creative Commons Attribution License from Guarnotta et al., 2019 [21].

	Group A (no=47)			Group B (no=53)		
	Baseline	After 48 months	Pa	Baseline	After 48 months	Pb
Anthropometric parameters						
BMI (kg/m^2^)	25.9 ± 5.3	28 ± 5.47	<0.001	27.6 ± 4.96	26.6 ± 5.06	0.002
WC (cm)	94 ± 13.5	100 ± 12	0.001	98 ± 13	94 ± 12	0.015
Systolic blood pressure (mmHg)	111 ± 14	121 ± 14	0.001	118 ± 15	115 ± 16	0.461
Diastolic blood pressure (mmHg)	68 ± 8	70 ± 10	0.251	71 ± 9	66 ± 9	0.031
Metabolic parameters						
Total cholesterol (mmol/L)	5.01 ± 0.75	5.07 ± 0.71	0.136	5.45 ± 1.31	4.72 ± 0.72	0.006
HDL cholesterol (mmol/L)	1.56 ± 0.54	1.45 ± 0.33	0.137	1.52 ± 0.48	1.58 ± 0.39	0.284
Triglycerides (mmol/L)	1.41 ± 0.76	1.48 ± 0.72	0.131	1.60 ± 0.76	1.47 ± 0.83	0.393
LDL cholesterol (mmol/L)	2.64 ± 0.9	3.03 ± 0.76	0.018	3.08 ± 0.99	2.46 ± 0.69	0.005
HbA1c (mmol/mol)	34.8 ± 22.8	43.1 ± 10.4	0.02	48.7 ± 16.9	39.1 ± 8.9	<0.001
Fasting glycemia (mmol/L)	5.45 ± 3.22	5.47 ± 2.57	0.96	5.65 ± 3.57	5.42 ± 3.51	0.549

Quinkler and Hahner [22] found that patients with primary adrenal insufficiency have increased mortality and morbidity due to their long-term use of glucocorticoid and mineralocorticoid replacement therapy. They defined that these patients with AI need to adjust the dose of their glucocorticoid treatment in cases of elevated stress so that they can cover the demand needed. Additionally, they make various recommendations to avoid adrenal crises, such as: being aware of precipitating factors like stress, illness, infections, fever, etc. It is also advantageous to teach patients about the importance of a glucocorticoid emergency kit at hand (including prednisolone rectal suppositories), though, in cases of diarrhea, a hydrocortisone intramuscular injection was found to be best if possibly self-administered in emergent situations.

Long-term use of oral glucocorticoids and their result in adrenal insufficiency 

Glucocorticoids are widely used anti-inflammatory drugs. Oral and intra-articular forms, as well as long-term use and high-dose treatment, have the most risk of inducing adrenal insufficiency [17]. Overman and colleagues conducted a study that evaluated the prevalence of oral glucocorticoid use in the United States, analyzing a group of 35209 subjects over the age of 20 from 1999 to 2008. Of those 35,209, 26,248 were interviewed, and 356 of them reported current use of an oral glucocorticoid; this would be 1.2% of the US general population at the time. They also reported that the highest usage was in female patients, and in general, in those >80 years old [23]. Also, of those who reported taking oral glucocorticoids, the mean duration of use was estimated to be over four years. This is because 65.2% had been receiving the medication for more than 90 days. 

Role of antifungals in causing adrenal insufficiency

Bornstein also found in 2009 that 1% of the general population is treated with long-term glucocorticoid regimes related to chronic diseases and inflammation [13]. He found an increase in cases of adrenal insufficiency among patients receiving antifungal therapies. This is important to this review because such medicines as ketoconazole (antifungals) interfere with glucocorticoid synthesis and therefore are also used in high doses to combat hypercortisolism. This also reinforces the importance of a complete medical history to see the different medications a patient is on and their anti-glucocorticoid effect.

Glucocorticoid use in children 

Few case reports discuss adrenal crises secondary to glucocorticoid use and even deaths in children. These were predominantly in children receiving inhaled corticosteroid (ICS) therapy like fluticasone or treatment for a significant ailment such as acute lymphoblastic leukemia [24, 25]. In the pediatric community, the ideal regimen is IV hydrocortisone given thrice daily in a dose ranging from 7-12 mg/m2/day. 

Prednisone, prednisolone, and dexamethasone are alternatives that can be used in adolescent patients in cases where tolerance with other glucocorticoids has been reached. The benefit of these is that they are long-acting glucocorticoids, which can be applied once a day. Oprea et al. found that the best way to prevent an adrenal crisis was by identifying the precipitating risk factors and simultaneously slowly tapering off medication to either base levels or to the where the medication was no longer needed [24]. Even though a taper hasn't been found to prevent adrenal insufficiency, it can help be more conscious of the effects and symptoms of the medication as it allows for recognizance of the hypothalamic-pituitary-adrenal axis recovery [3]. 

Discussion 

Exogenous glucocorticoids are one of the leading causes of secondary adrenal insufficiency, especially in prolonged treatment or shortly after abrupt withdrawal [26, 27]. The classic short Synacthen test (SST; 250μg of ACTH, intramuscular (IM) or intravenous (IV)) is considered the standard diagnostic method to detect adrenal insufficiency, with a sensitivity of 92% [28]. However, diagnosing adrenal insufficiency in patients receiving corticosteroids is not routine practice. The inconsistency between frequent biochemical diagnosis and reported clinical manifestation of adrenal suppression, which could be non-specific symptoms such as fatigue or abdominal pain and could be attributed to other causes, indicates that there is a significant potentially under-recognized population [29-32]. Many individuals only have biochemical signs of adrenal insufficiency, such as hyponatremia or hypoglycemia, until physiological stress accumulates into morbidities such as adrenal crisis and even mortality [33-37]. Adrenal crisis is an acute deterioration in health associated with hypotension, acute abdominal symptoms, and various laboratory abnormalities, which resolve after parenteral glucocorticoid administration. Even though the diagnostic investigation is well established, it can be challenging, especially in patients with secondary or tertiary adrenal insufficiency. A retrospective case-control analysis of the European Adrenal Insufficiency Registry (EU-AIR) reported an incidence of adrenal crises of around 6.5 cases per 100 patient-years for primary adrenal insufficiency (PAI) and 3.2 cases per 100 patient-years for central adrenal insufficiency [38]. In addition, many acute cases are discovered through emergency admission. Under emergency admission, it is relatively uneasy to detect adrenal insufficiency due to its non-specific symptoms, leaving patients in life-threatening medical circumstances [16, 30, 39, 40]. Therefore, research has called for increased awareness for physician and patient education on glucocorticoid-induced adrenal insufficiency [33, 41, 42].

The management of adrenal insufficiency has been challenging despite new developments in the recent past. In patients with primary adrenal insufficiency, the long-term use of glucocorticoids results in higher rates of mortality and morbidity when compared to patients without primary adrenal insufficiency and are on long-term glucocorticoid therapy [43]. Moreover, according to Donaldson et al. [35], dual-release hydrocortisone (DR-HC) has shown better metabolic changes than conventional glucocorticoids. The results show that long-term DR-HC reduces levels of lab results for HbA1c, BMI, waist circumference (WC), TC, and low-density lipoprotein cholesterol (LDL-C) after 48 months. Moreover, according to the renal function of the patient undergoing long-term glucocorticoid therapy, the dose of glucocorticoid can be adjusted, which helps to prevent adrenal crisis [35]. Dose alteration is also helpful in patients prone to having risk factors of adrenal crisis, like stress, fever, etc. Patients have to be counseled for monitoring glucocorticoid therapy regarding the total dosage per day, the number of doses, and the timeline for each dosage [44]. Involving the family and friends of the patient would also result in better patient compliance and immediate action during any emergency [45].

Individual prediction of adrenal insufficiency associated with oral glucocorticoid use is not yet accessible due to the wide range of glucocorticoid formulas, doses, and treatment duration. Prete and Bancos found out that patients previously taking exogenous glucocorticoids and presenting with an adrenal crisis or Cushingoid appearance has a very high risk of adrenal suppression due to glucocorticoid use [46]. It is reported that higher daily and cumulative doses of oral glucocorticoid use increase the risk of adverse adrenal outcomes and mortality [29, 47]. In adults, having a glucocorticoid regimen comparable with over 20 mg/day of prednisone for more than two weeks or over 5 mg of prednisone for more than three to four weeks can suppress the HPA axis [46]. In children, the daily dose is more than 2-3 mg/m2 of prednisone for over four weeks [48]. Of note, patients with inflammatory bowel disease taking more than 6 mg/day of oral budesonide for over eight weeks or on long-term prescriptions should be considered to have a higher risk of developing adrenal insufficiency [49, 50].

A meta-analysis by Broersen et al. [18] indicates that the absolute risk of developing adrenal insufficiency in patients taking oral glucocorticoids is 48.7%. This is higher than in patients who administer exogenous corticosteroids through other forms such as topical, inhalation, and nasal administration, which could lead to lower systemic corticosteroid levels compared to oral administration. They also found out that patients with hematological malignancies, rheumatic diseases, post-renal transplants, and patients receiving multiple administration modes of glucocorticoids have a higher incidence of developing adrenal insufficiency. This could be due to longer treatment duration and higher treatment doses of exogenous corticosteroids for those patients. The possible fact that patients receiving multiple administration modes of glucocorticoids have a higher incidence of developing adrenal insufficiency could also be seen in studies by Brennan et al. [51] and Mortimer et al. [52] on asthma patients, which report that the predictive prevalence of adrenal insufficiency is higher in patients receiving both inhaled corticosteroid therapy and maintenance use of oral corticosteroids. Other risk factors of glucocorticoid-induced adrenal insufficiency include the mode and timing of glucocorticoid administration and drug interactions [53]. Multiple daily split doses of oral glucocorticoids and bedtime administration could increase the risk of adrenal suppression by affecting circadian ACTH release [54-56]. In contrast, high-dose pulse therapy and alternate-day administration enable the HPA axis to recover and are associated with reduced incidence of adrenal insufficiency [46, 57-59]. It should be noted that concomitant use of systemic glucocorticoids with cytochrome P450 3A4 (CYP3A4) inhibitors, such as ritonavir and antifungal drugs, could decrease the metabolization of synthetic glucocorticoids, like dexamethasone and prednisolone, by CYP3A4, which could put patients at higher risk of developing adrenal insufficiency [60-64]. 

It is noted that variation within individual susceptibility to glucocorticoid-induced adrenal insufficiency should be considered with careful clinical judgment. Studies have shown that gene polymorphisms among mineralocorticoid and glucocorticoid receptors could affect an individual's sensitivity and response to glucocorticoids, which would result in higher or lower rates of hypercortisolism or glucocorticoid resistance [65-72]. A genome-wide association study by Hawcutt et al. also reports that single nucleotide polymorphisms within the platelet-derived growth factor D (PDGFD) gene locus could be associated with an increased risk of developing corticosteroids-induced adrenal suppression in children with asthma and adult with chronic obstructive pulmonary disease (COPD) [73]. These discoveries might help identify patients benefitting from alternative treatments to avoid unnecessary adverse effects. During the discontinuation of GCs therapy, clinicians should anticipate glucocorticoid withdrawal syndrome, symptoms of adrenal insufficiency, and a possible relapse of the original disease prior to the treatment [74, 75]. A Danish study shows that signs of adrenal suppression like hypotension, hyponatremia, hypoglycemia, and gastrointestinal symptoms in the study population reached their peak in the third month after ceasing long-term oral glucocorticoid treatment [76]. This marks the importance of glucocorticoid taper. When assigning a glucocorticoid tapering plan, it is important to consider patients dosage and duration of glucocorticoid use, health status, basic cortisol levels, previous performance during tapering, withdrawal syndromes, and chances of underlying disease relapse [74, 77, 78]. However, evidence on tapering regimens and therapy for the HPA axis recovery is inconclusive. It is suggested that no taper is needed for oral glucocorticoid therapy that is less than two weeks due to the low risk of HPA axis suppression. For patients receiving prednisolone over 20-40 mg/day, a rapid glucocorticoid taper of 5-10 mg weekly until 20 mg/day is recommended. A weekly to monthly slow taper of 1-2.5 mg afterward is needed for most patients [46, 79]. 

In pediatric diseases, oral glucocorticoids are widely used for their anti-inflammatory and immunosuppressive effects [3, 25, 80-83]. Research has shown biochemical signs and clinical symptoms of adrenal insufficiency or withdrawal syndromes, including fever, vomiting, anorexia, headache, lethargy, arthralgia, myalgia, and hypotension after discontinuation of therapy despite tapering off glucocorticoid doses [4, 33, 81, 84, 85]. Duration of adrenal insufficiency after glucocorticoid exposure is reported to be less in patients receiving short-term treatment. Patients who develop adrenal insufficiency after receiving one month of glucocorticoid therapy often recover in weeks to months [85-88]. Patients with high-dose or long-term glucocorticoid use, lower body mass index, or iatrogenic Cushing's syndrome may need a longer time to recover their adrenal function [74, 89]. Prospective studies by Einaudi et al. [81] and Rix et al. [25] evaluate children with acute lymphoblastic leukemia (ALL) who received four to five-week induction therapy, including high-dose oral prednisone or dexamethasone, followed by a nine-day tapering phase. Patients' adrenal function returned to baseline within several weeks after treatment. Another study by Ahmet et al. [85] shows that 54.8% of the studied adolescent patients with rheumatic conditions presented with signs of adrenal insufficiency after more than four weeks of daily oral glucocorticoids use, along with intermittent systemic and non-systemic forms of glucocorticoid therapy, with a four-week taper. Their adrenal suppression persisted over seven months to a year. These studies above highlight the necessity of cortisol levels monitoring and steroid coverage used in stress episodes after cessation of glucocorticoid therapy, even tapering the dose. 

Moreover, Goldbloom et al. [33] reported significant complications of adrenal suppression in asthmatic children receiving intermittent oral or intranasal glucocorticoids in addition to long-term inhaled corticosteroids. They suggest clinicians often revisit glucocorticoid doses to be sure patients are under the lowest effective dosage, and they call for proactive screening for adrenal suppression in patients with higher risks. Harel et al. [90] and Ahmet et al. [91] also report an increased incidence of adrenal insufficiency in children taking one month of oral viscous budesonide and swallowed fluticasone for eosinophilic esophagitis (EoE). Therefore, weeks of stress steroids after therapy discontinuation is advised to prevent a likely adrenal crisis. The diagnosis of adrenal crisis can be delayed until there is an overlapping illness, such as a severe infection like sepsis or any other acute stress [9]. When an adrenal crisis is present, although diagnosis is necessary, there is no need for an immediate investigation to confirm adrenal insufficiency. Still, treatment should be initiated without delay as soon as clinically suspected. Adequate awareness among patients and medical care teams for managing adrenal insufficiency and adrenal crisis is necessary to improve clinical outcomes [28]. Although guidelines for diagnosing and managing primary or secondary adrenal insufficiency and adrenal crisis have been proposed, there are also guidelines specifically for glucocorticoid-induced adrenal insufficiency [45, 92-95]. The extensive formulation, duration, range of dosage of glucocorticoid treatment, and the heterogeneity of existing literature form the natural limitation for explicit guidance. More emphasis will likely be put on educating patients at risk of adrenal insufficiency and those more prone to an adrenal crisis. Services including therapy counseling and educating patients on signs and symptoms of over-replacement and under-replacement of glucocorticoids would be beneficial. 

Considering the wide prevalence of long-term oral glucocorticoid use among the population and possible excessive cumulative glucocorticoid exposure due to frequent concomitant use with other modes of administering glucocorticoid therapy, it is crucial to identify risk factors that may lead to glucocorticoid-induced adrenal insufficiency and potential development of the life-threatening adrenal crisis. 

## Conclusions

Oral glucocorticoids, when given for long periods, put patients at risk of developing secondary adrenal insufficiency due to suppression of the HPA axis. The prevalence of long-term oral glucocorticoid use is 1% in the general population and increases with age and among women. The absolute risk of having adrenal insufficiency in patients taking oral glucocorticoids is 48.7%, which is much higher than with inhaled, nasal and topical administration. Daily doses of over 20 mg/day in adults for more than two weeks, and in children, doses of more than 2-3 mg/kg/m2 prednisone for more than four weeks can cause HPA axis suppression. Recovery is quick if glucocorticoids have been used for under 10-14 days. Prolonged time for recovery of adrenal function is seen with a higher dosage, longer duration, lower BMI, or iatrogenic Cushing's syndrome. Multiple daily doses and bedtime administration increase adrenal suppression risk by a more significant amount than when given in an alternate-day administration. Low-dose ACTH test was found to be excellent, with high sensitivity for the HPA axis assessment. One must customize tapering by considering dosage, duration, cortisol levels, previous taper performance, and risk of disease relapse for speedy HPA axis recovery. Therapy counsel, patient education on steroid coverage for periods of stress, illness, or surgery, and research on prevention, diagnosis, and management of glucocorticoid-induced adrenal insufficiency are crucial. Substantial individual variation, diagnosis, risks, prognostic factors, and factors affecting morbidity need to be further researched and a consensus developed to identify appropriate tapering strategies and to prevent and manage adrenal insufficiency for patient benefit.
